# Replacing Harmful Flame Retardants with Biodegradable Starch-Based Materials in Polyethylene Formulations

**DOI:** 10.3390/polym15204078

**Published:** 2023-10-13

**Authors:** Bárbara O. Carvalho, Luís P. C. Gonçalves, Patrícia V. Mendonça, João P. Pereira, Arménio C. Serra, Jorge F. J. Coelho

**Affiliations:** 1 Centre for Mechanical Engineering, Materials, and Processes, Advanced Production and Intelligent Systems (ARISE), Department of Chemical Engineering, University of Coimbra, Rua Sílvio Lima-Polo II, 3030-790 Coimbra, Portugal; 2 Componit Lda, Estr. Real 3, 2070-621 Vila Chã de Ourique, Portugal

**Keywords:** starch, flame retardant, biodegradable polymer, polyethylene, fire behavior

## Abstract

The addition of toxic flame retardants to commercially available polymers is often required for safety reasons due to the high flammability of these materials. In this work, the preparation and incorporation of efficient biodegradable starch-based flame retardants into a low-density polyethylene (LDPE) matrix was investigated. Thermoplastic starch was first obtained by plasticizing starch with glycerol/water or glycerol/water/choline phytate to obtain TPS-G and TPS-G-CPA, respectively. Various LDPE/TPS blends were prepared by means of melt blending using polyethylene graft maleic anhydride as a compatibilizer and by varying the content of TPS and a halogenated commercial flame retardant. By replacing 38% and 76% of the harmful commercial flame retardant with safe TPS-G-CPA and TPS-G, respectively, blends with promising fire behavior were obtained, while the limiting oxygen index (LOI ≈ 28%) remained the same. The presence of choline phytate improved both the charring ability and fire retardancy of starch and resulted in a 43% reduction in fire growth index compared to the blend with commercial flame retardant only, as confirmed by means of cone calorimetry. Standard UL 94 vertical tests showed that blends containing TPS exhibited dripping behavior (rated V2), while those with commercial flame retardant were rated V0. Overall, this work demonstrates the potential of starch as a natural flame retardant that could reduce the cost and increase the safety of polymer-based materials.

## 1. Introduction

Polyethylene (PE) is one of the most widely used polyolefins in the global plastics market (around 34%) [1] and is available in three main grades: low-density polyethylene (LDPE), linear low-density polyethylene (LLDPE) and high-density polyethylene (HDPE) [2,3]. The popularity and wide range of applications of PE are related to its good mechanical properties, ease of processing, low toxicity, and electrical insulation properties, at an affordable price [4]. LDPE, for example, is one of the most widely used polymers for electrical insulation because it combines suitable mechanical properties and good electrical properties, namely low permittivity, and high electrical breakdown strength [1]. According to the European Commission, the LDPE market was valued at USD 4 billion in 2020 [5]. The market is forecast to grow at a 3% CAGR (compound annual growth rate) from 2023 to 2028 [6]. However, due to its long aliphatic chains, LDPE is highly flammable and cannot tolerate temperatures above 70 °C, which limits its application in many sectors, such as transportation, electronics, among others [7,8]. Therefore, the addition of flame retardants (FRs) to LDPE is a simple strategy to reduce the flammability of this polymer, increase its survival time in the event of fire and ensure the safety levels required for each application. There are several types of commercially available FR, such as compounds based on phosphorus, borate, inorganic hydroxides, silicon, nitrogen, and halogen-containing molecules [9,10], that can be used in LDPE formulations, and these are usually incorporated into the polymer during processing. Information on the mechanism of action and application of these FRs in LDPE can be found in a recent review article [1]. The commercially available FRs used in LDPE are usually halogenated FR in combination with antimony trioxide (ATO, Sb_2_O_3_), magnesium hydroxide (MDH, Mg(OH)_2_), or aluminum hydroxide (ATH, Al(OH)_3_) compounds. Despite their good flame-retardant performance, halogen containing FRs are not good candidates from an environmental point of view due to their high toxicity, and some of them were banned a few decades ago [11]. In view of current environmental concerns, it is therefore highly desirable to develop new halogen-free FRs that are both safe and can be obtained from renewable and sustainable sources [12,13]. Several biobased materials, such as deoxyribonucleic acid (DNA) [14], β-cyclodextrin [14], organic phosphorus compounds [15], phytic acid [7], polydopamine or tannic acid [12], have been proposed as environmentally friendly FRs. The use of various natural polymers, such as starch, wood flour, chitosan, lignin, and others as FRs has also been demonstrated with the aim of improving both the biodegradability and flame retardancy of polymer materials [13].

Using starch as an FR is a wise and promising option because this natural polymer is safe, abundant, and inexpensive. Starch from various sources has been successfully blended with LDPE via extrusion, resulting in blends with inferior mechanical properties in comparison with neat LDPE [16]. In fact, plasticizing starch with different molecules, namely water, polyols, or choline phytate (CPA), to give the so-called thermoplastic starch (TPS) is necessary to provide some workability and mechanical properties to starch-based materials [7,17,18]. However, the incorporation of TPS into LDPE results in materials with poor mechanical properties. This is due to the incompatibility between the highly hydrophilic character and strong intra- and inter-molecular hydrogen bonding of the starch, and the hydrophobic nature of the polyolefin [19]. A straightforward and simple strategy to improve the miscibility of TPS/LDPE blends is to use commercially available compatibilizers, such as PE grafted with glycidyl methacrylate (PE-*g*-GMA) or LDPE-grafted maleic anhydride (LDPE-*g*-MA) [20]. Unfortunately, all reported work uses starch/TPS as a filler for LDPE to improve the biodegradability of this polyolefin. To the best of our knowledge, the use of starch as a flame retardant in LDPE formulations has never been reported.

The aim of this work was to develop a new starch-based flame retardant that can be used in PE formulations. In this way, it will be possible to obtain a low-cost and partially biodegradable product and reduce or eliminate the need for harmful flame retardants. For this purpose, various LDPE/TPS blends with different proportions and compositions of TPS were prepared by means of melt blending. The effect of combining the biobased FRs developed in this work with a commercial halogenated flame retardant in LDPE blends was also investigated. The materials were analyzed using scanning electron microscopy (SEM), tensile tests, thermogravimetric analysis (TGA), the limiting oxygen index (LOI), flammability (UL 94) and cone calorimetry.

## 2. Materials and Methods

### 2.1. Materials

Choline chloride (>99.0%, Acros Organics, Geel, Belgium, Germany), ethanol absolute (>99.5%, PanReac AppliChem ITW Reagents, Darmstadt, Germany), deuterated chloroform (CDCl_3_, Eurisotop, Saarbrücken, Germany), deuterated dimethyl sulfoxide (*d*_6_-DMSO, Eurisotop, Saarbrücken Germany), deuterated water (D_2_O, Eurisotop, Saarbrücken Germany), glycerol (>99%, Merck, Darmstadt, Germany), phytic acid (ca. 50% in water, ca. 1.1 mol/L, TCI Europe, Zwijndrecht, Belgium, Germany) and sodium hydroxide (NaOH, José Manuel Gomes dos Santos, LDA, Odivelas, Portugal) were used as received.

IsoAdditive FR L069 flame retardant (brominated-based additives in LDPE carrier) was kindly supplied by Isolago (Aveiras de Baixo, Portugal) and used as received.

Potato starch, low-density polyethylene (LDPE) with a melting temperature of 140 °C (Maxxam^TM^) and polyethylene-*graft*-maleic anhydride (PE-*g*-MA, Orevac OE825, SK-fp) were kindly supplied by Componit (Vila Chã de Ourique, Portugal) and used as received.

### 2.2. Techniques

TPS and LDPE/TPS blends were prepared by means of melt blending using a laboratory mixer HAAKE^TM^ Polylab^TM^ QC (Thermo Scientific^TM^, Waltham, MA, USA).

We recorded 400 MHz ^1^H nuclear magnetic resonance (^1^H NMR) spectra of the compounds using a Bruker Avance II 400 MHz Spectrometer (Bruker Biospin, Wissemboug, France) with a 5 mm TIX triple resonance detection probe.

Fourier transform infrared (FTIR) spectra were obtained in the range of 4000 to 750 cm^−1^, with 4 cm^−1^ spectral resolution and 64 accumulations, at room temperature using an Agilent Technologies Carey 630 spectrometer (Agilent, Santa Clara, CA, USA) equipped with a golden gate single reflection diamond ATR.

The mechanical properties of the blends were accessed via tensile tests using a Chatillon TCD100 machine (Transcat, Rochester, NY, USA) at 5 mm/min speed and a load cell of 5 kN, with at least 5 specimens (10 ± 0.5 mm × 70 ± 0.5 mm).

Samples for scanning electron microscopy (SEM) analysis were prepared using the fraction surface of the tensile test’s specimens. The fracture surface was coated with gold and analyzed in a Field Emission SEM ZEISS MERLIN Compact/VP Compact Gemini II (Zeiss, Madrid, Spain).

Vertical flammability assays were conducted by AIMPLAS (Spain) following the UL 94:2013 standard [21].

LOI assays were conducted by AIMPLAS (Spain) using the Netzsch-Taurus Oxygen (Netzsch, Bobingen, Germany) system following the standard UNE-EN ISO 4589-2:2017 [22]. Average sample thickness (mm): 1.5; average sample width (mm): 10.

Cone calorimeter tests were conducted by AIMPLAS (Spain) using the FTT ICONE CLASSIC machine (Fire Testing Technology, East Grinstead, UK) and following the standard EN ISO 5660-1:2015 [23]. Conditions: exhaust flow rate (L/s): 0.24; irradiance (kW/m^2^): 50; orientation: horizontal; separation (mm): 25; wire grid: No; N of specimens: 3; average sample thickness (mm): 1.5; pre-conditioning 48 h at 23 ± 2 °C, 50 ± 10%RH; environmental conditions: 23 ± 2 °C; 50 ± 10%RH

Thermogravimetric (TG) analyses were carried out using a NETZSCH STA 44F5 (Netzsch, Selb, Germany), from 20 °C to 500 °C, at a heating rate of 10 °C·min^−1^, under nitrogen purge flow (sample weight in the range of 5 to 10 mg).

### 2.3. Procedures

#### 2.3.1. Synthesis of Choline Phytate

CPA was prepared according to the procedures described in the literature [24]. First, choline hydroxide ([Chol][OH]) was prepared using the ion exchange method. For this purpose, NaOH (28.0 g, 0.7 mol) and choline chloride ([Chol][Cl]), 97.7 g, 0.7 mol) were dissolved in 375 mL and 250 mL of absolute ethanol, respectively. The solutions were mixed and stirred at room temperature for 1 h. The formed white precipitate (sodium chloride) was removed via vacuum filtration, and the solution of [Chol][OH] in ethanol was obtained. Phytic acid (77.2 g, 0.117 mol) was then dissolved in 100 mL of absolute ethanol. The phytic acid solution was added to the [Chol][OH] solution and stirred for 1 h at 30 °C. The ethanol was removed under reduced pressure. Finally, CPA was obtained after drying under reduced pressure at 80 °C for 24 h. The chemical structure of CPA was confirmed through ^1^H NMR spectroscopy (Figure S1).

#### 2.3.2. Preparation of TPS

Potato starch was plasticized by two different methods, giving TPS-G (plasticized with glycerol and water) and TPS-G-CPA (plasticized with glycerol, water and CPA). Table 1 shows the composition of each TPS type.

All compounds were placed in a plastic flask and mixed manually with a spatula. The flask was sealed and the mixture was allowed to stand overnight. The mixture was then processed via melt mixing at 120 °C (TPS-G) or 140 °C (TPS-G-CPA) for 5 min, at 100 rpm.

#### 2.3.3. Preparation of LDPE/TPS Blends

To prepare LDPE/TPS blends, LDPE, TPS, PE-*g*-MA compatibilizer, and a commercial flame retardant (FR L069, if used) were added to a mixing chamber. The mixtures were processed at 140 °C with a rotation speed of 100 rpm for 5 min. The obtained mixtures were cut into small pieces by hand, placed between two silicone squares and pressed in a Carver^®^ hydraulic press under 0.5 bar at 140 °C for 5 min, followed by cooling at room temperature. The compounds were produced in the form of sheets with a thickness of approx. 5 mm.

## 3. Results and Discussion

### 3.1. Preparation and Characterization of the Blends

**Figure 1:**
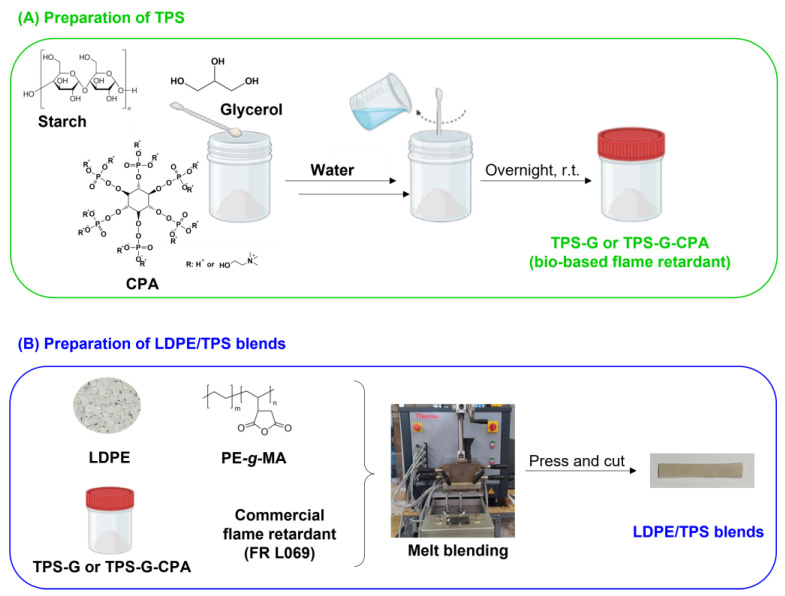
General scheme of the preparation of (**A**) biobased FR, TPS-G and TPS-G-CPA and (**B**) LDPE/TPS blends.

Starch is an inexpensive and naturally occurring polysaccharide that exhibits charring properties, and has been considered as a potential biobased flame retardant for various different applications [25,26]. In this work, we investigated the possibility of replacing a commercial flame retardant (FR L069) used in LDPE formulations with a starch-based flame retardant, thereby reducing production costs and increasing both the safety and biodegradability of the final material. Initially, potato starch was plasticized with water and glycerol to form TPS-G, to obtain starch-based LDPE blends with adequate plasticity. To improve the efficacy of the developed biobased flame retardants, starch was also plasticized in the presence of CPA (TPS-G-CPA), as this compound has been described as both a flame retardant and a starch plasticizer [24]. It is worth noting that CPA is also a biobased compound derived from phytic acid, which is produced by plants to store phosphorus. To increase the compatibility between hydrophobic LDPE and hydrophilic TPS g or TPS-G-CPA [24] and ensure the mechanical properties of the blends, a reactive PE-*g*-MA compatibilizer was also incorporated into the formulation during the melt blending process (Figure 1). This copolymer is commonly used in the preparation of PE/TPS blends because the maleic anhydride groups can react with the hydroxyl groups of starch, while the PE segment has an affinity for the LDPE matrix [19].

Various LDPE/TPS blends compatibilized with 5 wt% PE-*g*-MA (optimized content) were prepared by means of melt blending at 120 °C and 140 °C, respectively, when TPS-G or TPS-G-CPA was used as the biobased flame retardant (Table S1). Considering that the objective of this work was to replace a commercial harmful flame retardant in LDPE formulations with a biodegradable starch-based one, preliminary tests were conducted in the laboratory mimicking the conditions of the UL 94 standard to evaluate the potential of the prepared blends as flame-retardant and self-extinguishing materials. For this purpose, 7 cm × 1 cm samples of LDPE/TPS-G(-CPA) blends were positioned vertically above a Bunsen burner flame and exposed to the flame (approximately 1 cm) for 10 s or until combustion started (if it occurred in less than 10 s). This time was recorded as the ignition time. The flame source was then removed and the time until the flame was extinguished was recorded as the first quenching time. The results (Figure S2) show that all samples did not extinguish the flame on their own, as they burned completely. However, it is worth noting that flame retardancy increased with increasing starch content in the blends, suggesting that this biomolecule indeed has the potential to be used as a flame retardant for LDPE. Interestingly, the combination of starch and CPA improved the flame retardancy of the materials, as shown by the 2.5 times higher quenching time of the blend 47.5 LDPE/47.5 TPS-G-CPA compared to 47.5 LDPE/47.5 TPS-G. Unfortunately, dripping was observed in all samples, which can be considered detrimental in terms of flame resistance, as this event may represent an additional ignition source or a process of flame propagation during a fire [27].

Although not complete, the replacement of any percentage of a harmful FR with those based on TPS in the formulations of PE without adverse effects on the performance of the material is highly desirable from the standpoint of waste management and environmental protection. Therefore, the effect of incorporating a halogenated commercial flame retardant (FR69) into the LDPE/TPS-G(-CPA) blends prepared in this work was investigated. Table 2 shows the composition of the LDPE/TPS-G(-CPA)/FR69 blends studied, where the total flame retardant content was set at 50 wt% and the starch-based flame retardant content ranged from 9.5 to 38 wt%.

**Figure 2:**
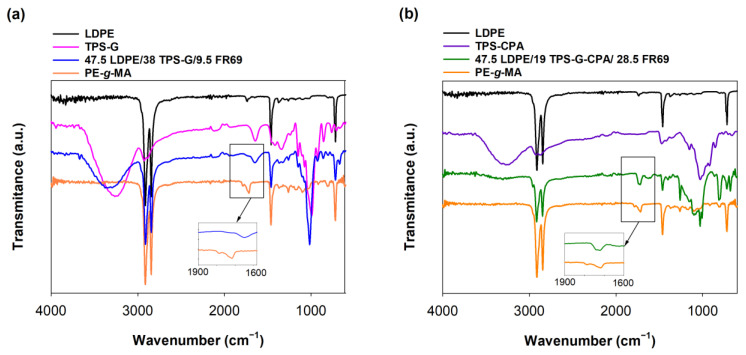
FTIR spectra of LDPE (black line), TPS-G (pink line), 47.5 LDPE/38 TPS-G/9.5 FR69 (blue line), PE-*g*-MA (orange line), TPS-G-CPA (purple line) and 47.5 LDPE/19 TPS-G-CPA/28.5 FR69 (green line). (**a**) LDPE/TPS-G-based blend and (**b**) LDPE/TPS-G-CPA-based blend.

FTIR analysis (Figure 2) confirmed the chemical structure of LDPE, namely the bands at 2918 cm^−1^, 2851 cm^−1^, 1468 cm^−1^ and 718 cm^−1^, corresponding to CH_2_ asymmetrical stretching, CH_2_ symmetrical stretching, bending deformation and rocking deformation, respectively [28]. The FTIR spectrum of TPS-G (Figure 2a) was also consistent with the literature and showed the expected signals of *C−O* stretching at 920 cm^−1^, 1022 cm^−1^ and 1148 cm^−1^, bound water at 1648 cm^−1^, hydroxyl groups at 3277 cm^−1^, *CH* stretching at 2914 cm^−1^ and glycerol at 1423 cm^−1^ [29]. TPS-G-CPA showed a similar FTIR spectrum (Figure 2b) to that of TPS-G. The reactive compatibilization between PE-*g*-MA and TPS-G was confirmed by the disappearance of the symmetric and asymmetric maleic anhydride groups of PE-*g*-MA at 1714 cm^−1^ and 1791 cm^−1^, respectively, in the FTIR spectrum of the blend (Figure 2a). This indicates successful esterification between the anhydride rings of the compatibilizer and the hydroxyl groups of TPS [30]. Compatibilization appeared to be less effective for the blends containing TPS-G-CPA, as indicated by the remaining maleic anhydride groups of PE-*g*-MA at 1714 cm^−1^ in the FTIR spectrum of the blend (Figure 2b).

**Figure 3:**
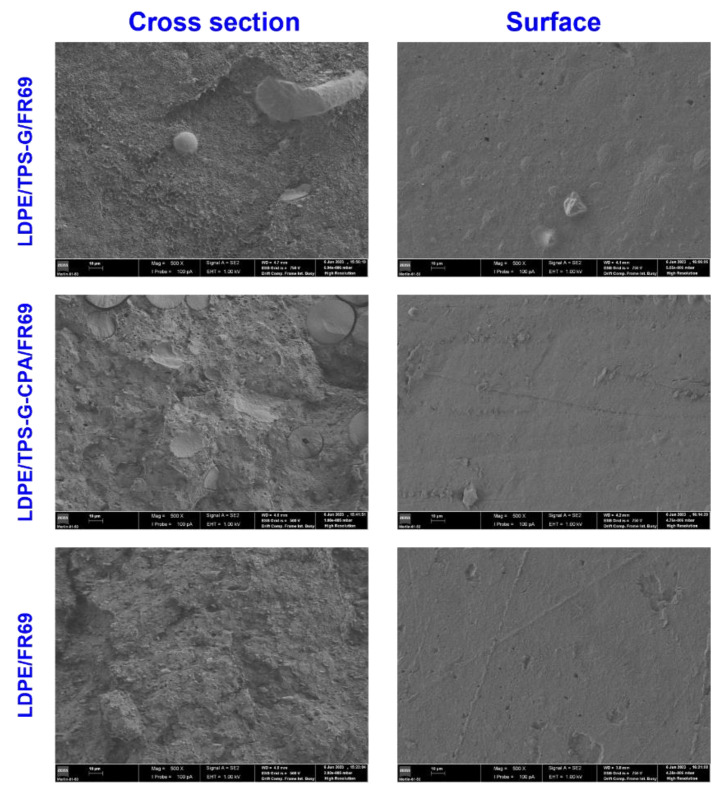
SEM images of the cross section and surface of LDPE/TPS/FR 69 blends, 1 kV and ×500: 47.5 LDPE/38 TPS-G/9.5 FR 69 (**top**); 47.5 LDPE/ 19 TPS-G-CPA/28.5 FR69 (**middle**) and 50LDPE/50FR 69 (**bottom**). Analysis conditions: EHT = 1.00 kV; Magnification = 500×.

To further evaluate the compatibility of LDPE/TPS/FR69 blends, representative samples were analyzed using SEM. Images of the surface of the blends (Figure 3) showed that both the control sample (LDPE/FR69 blend) and the blend with TPS-CPA had a similar appearance, while the blend with TPS-G had a rougher surface. However, all samples exhibited a smoother surface than that of non-compatibilized LDPE/TPS reported in the literature [31,32], confirming the role of PE-*g*-MA as an enhancer of the interaction between LDPE and TPS. Images of the fractured surfaces (cross section in Figure 3) confirm the FTIR results, indicating that the compatibilization of blends containing TPS-G-CPA was less effective, as indicated by the small gaps between a minority of TPS-G-CPA particles and the LDPE matrix. On the other hand, the sample containing TPS-G appeared to have a continuous phase between the starch particles and the LDPE, indicating better compatibilization, even at 40 wt% TPS-G compared to 19 wt% TPS-CPA.

### 3.2. Mechanical Analysis

**Figure 4:**
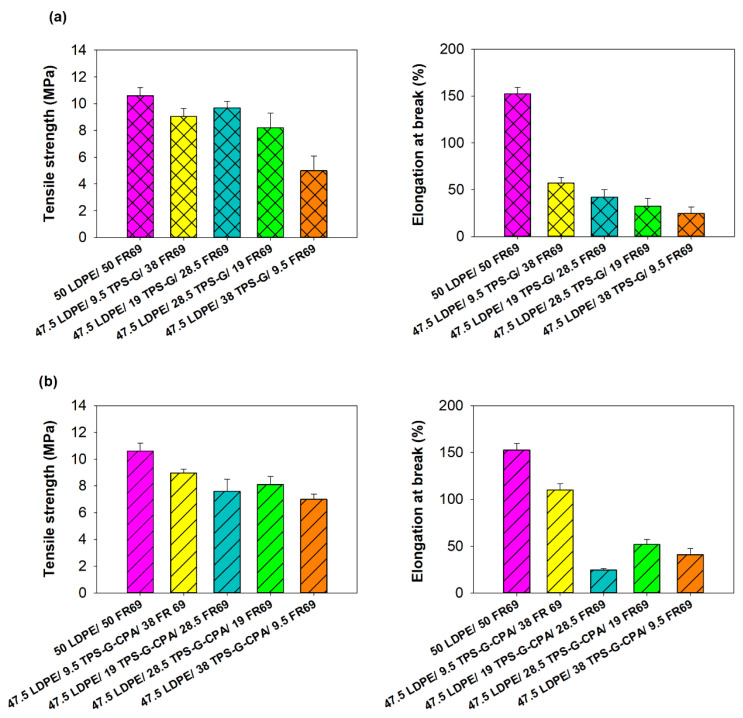
Tensile strength and elongation at break of (**a**) LDPE/TPS-G/FR69 blends and (**b**) LDPE/TPS-G-CPAFR69 blends. LDPE/FR69 was used as a reference material.

The incorporation of starch into LDPE is expected to result in a loss of mechanical properties of the polyolefin [18]. To investigate the extent of this event, LDPE/TPS-G(-CPA)/FR69 blends with different starch contents were subjected to tensile testing. The results shown in Figure 4 indicate that blends with a TPS-G(-CPA) content of up to 30 wt% generally exhibit similar tensile strength to LDPE/FR69, which was used as the reference material. However, as expected, elongation at break decreased dramatically with increasing TPS-G(-CPA) content in the blends. Interestingly, the results also confirmed the plasticizing effect of CPA, as the blends containing this molecule (TPS-G-CPA) generally exhibited higher elongation at break than the blends containing TPS plasticized only with glycerol and water (TPS-G) (Figure 4).

### 3.3. Fire Behavior

**Figure 5:**
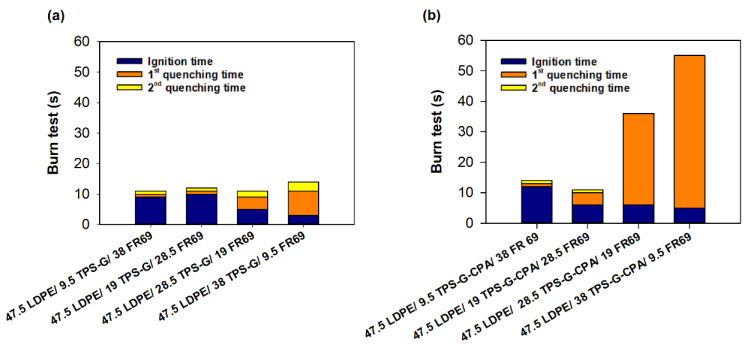
Preliminary burning tests conducted in the laboratory and mimicking the UL94 test for (**a**) LDPE/TPS-G blends and (**b**) LDEP/TPS-G-CPA blends. LDPE with a commercial flame retardant (76 LDPE/24 FR 69) was used as a reference material.

Preliminary flammability tests were conducted to evaluate the potential of LDPE/TPS-G(-CPA)/FR69 blends as flame retardant materials. The tests were conducted in the same manner as previously described for LDPE/TSP-G(-CPA) blends, but this time the materials were re-ignited with a flame after the initial quenching, and the time until the flame was extinguished was recorded as the second quenching time. Compared to LDPE/TPS-G(-CPA) blends (Figure S2), LDPE-based blends containing both commercial and starch-based flame retardants exhibited higher flame resistance, as reflected by higher ignition times and lower extinction times (Figure 5). In fact, LDPE/TPS-G(-CPA)-based samples with up to about 30 wt% starch-based flame retardant showed very impressive results compared to the reference (LDPE/FR 69) (Figure 5a). At higher levels of TPS-G, the samples burned longer but still self-extinguished, which is very encouraging. Unexpectedly, the same behavior was not observed with the mixtures containing TPS-G-CPA (Figure 5b), which exhibited a longer first quenching time but were unable to support the second burn cycle. Investigation of this behavior is beyond the scope of this paper, and the results will be published elsewhere.

**Figure 6:**
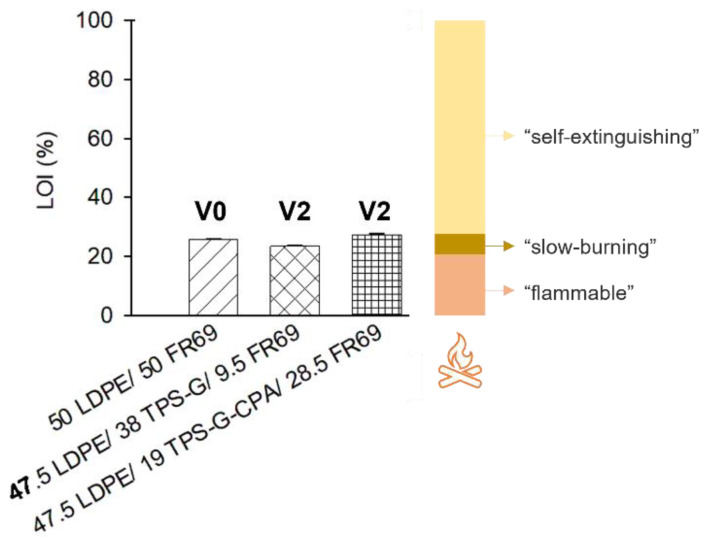
LOI and UL 94 rate (V0, V2) of selected LDPE/flame retardant blends. V0: Burning stops within 10 seconds on a vertical part allowing for drops of plastic that are not inflames; V2: Burning stops within 30 seconds on a part allowing for drops of vertical flammable plastic.

Blends with higher TPS content and higher flame retardancy in preliminary burning tests (47.5 LDPE/38 TPS-G/9.5 FR69 and 47.5 LDPE/19 TPS-G-CPA/28.5 FR69) were selected for further characterization and evaluation of their flame retardancy. LDPE with 50 wt% commercial flame retardant (50 LDPE/50 FR 69) was used as a reference material. The LOI can provide information about the relative flammability of polymers, as it indicates the minimum percentage (vol%) of oxygen in the atmosphere that can sustain a flame on a material. Therefore, the higher the flammability of the material, the lower the LOI value. It is noteworthy that the samples in which a portion of the commercial flame retardant was replaced with the starch-based products developed in this work have a similar LOI value to the reference material that contained only the commercial flame retardant (Figure 6). All tested blends are almost as good as “self-extinguishing” materials (LOI > 28%) [33], i.e., with good flame retardancy. Vertical UL 94 standard tests were conducted to evaluate the flammability of the LDPE-based blends. While LDPE containing the commercial flame retardant was rated V0 (self-extinguishing within 10 s with no dripping), both LDPE blends containing the bio-based and commercial flame retardant showed poorer results, as these samples took longer to self-extinguish and exhibited dripping (V2 rate, Figure 6).

The fire behavior of LDPE/flame retardant blends was evaluated using cone calorimetry, which mimics realistic fire conditions and provides several valuable parameters, particularly the rate of heat release rate during combustion. To increase the flame resistance of LDPE/flame retardant blends and reduce flame spread, it is desirable to have a low total heat release (THR) or a low peak of heat release rate (pHRR) [34]. The results presented in Table 3 show that both blends containing TPS have similar THR values, but they are about 30% higher than those of the reference material. Nevertheless, it is interesting to note that the pHRR value was similar for all LDPE/flame retardant blends (around 650 kW/m^2^). The values obtained for time to ignition (TTI) values in Table 3 show that the samples containing TPS started to burn two times faster than the samples containing only the commercial flame retardant. The results also show that the content of effective combustion components in LDPE could be reduced by using the biobased TPS-G-CPA flame retardant, as evidenced by the lower effective heat of combustion (EHC) of the corresponding LDPE blend compared to the reference material and the blend containing TPS-G. The propensity for fire development can be evaluated using the maximum average rate of heat emission (MARHE), which is the cumulative heat emission during the cone calorimetry test divided by time [35]. Both samples with the biobased FR had a higher MARHE (8% and 24%, respectively) than the reference material. However, it is important to note that the fire resistance of materials is affected by several parameters mentioned above. One way to evaluate overall fire safety is to determine both the fire performance index (FPI = TTI/pHRR) and the fire growth index (FGI = pHRR/tpHRR) [36]. For low fire risk and high safety levels, it is desirable to maximize FPI and minimize FGI. The results presented in Table 3 show that the FPI was reduced by 43% when 76% and 38% of the commercial flame retardant was replaced with TPS-G and TPS-G-CPA, respectively. However, it is noteworthy that the blend in which 38% of the commercial flame retardant was replaced with TPS-G-CPA showed a significant reduction (43%) in FGI, indicating that the blends may contribute to lower fire spread than the reference material.

### 3.4. Thermal Analysis

**Figure 7:**
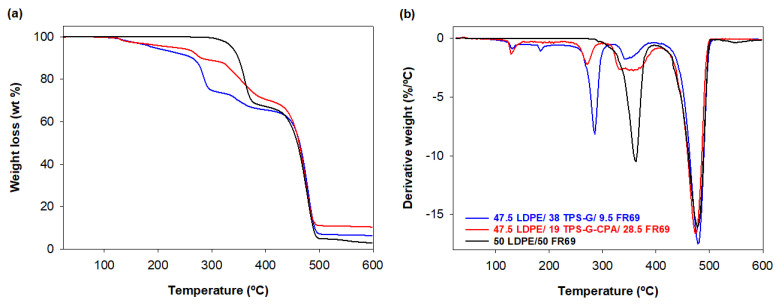
Thermogravimetric curves (**a**) TG and (**b**) DTG of the blends 47.5 LDPE/38 TPS-G/9.5 FR69 and 47.5 LDPE/19 TPS-G-CPA/28.5 FR69 under nitrogen atmosphere.

The thermal stability of the blends was investigated via TGA, and the TG and DTG curves are shown in Figure 7. The weight loss profile (Figure 7a) shows that the decomposition of the blends followed a similar and expected four-step decomposition process, with (i) moisture loss (*T* ≈ 128 °C), (ii) degradation of glycerol (*T* ≈ 270 °C), (iii) degradation of starch, CPA, and FR69 (*T* ≈ 350 °C), and (iv) degradation of LDPE (*T* ≈ 475 °C) [24,37]. The blend containing starch plasticized in the presence of CPA exhibited higher thermal stability than the blend containing TPS-G, as judged by both *T*_5wt%_ and *T*_10wt%_ of the samples (Table 4). In addition, the char residue of the mixture containing 19 wt% TPS-G-CPA was 10.5% at 600 °C, while the residue of the mixture containing about twice the amount (40 wt%) of TPS-G was 6.5% (Table 4). These results indicate that CPA can indeed improve the charring ability and flame retardancy of starch. Similar observations have been made in the literature [24]. As expected, the reference material (50 LDPE/50 FR69) exhibited higher thermal stability than the starch-containing blends.

## 4. Conclusions

In this work, the possibility of using starch as a natural flame retardant for LDPE was investigated. Different blends of LDPE/TPS g and LDPE/TPS-G-CPA were prepared by means of melt blending in the presence of PE-*g*-MA as a compatibilizer. The results of FTIR spectroscopy and SEM analysis showed that there was good compatibilization between starch and LDPE. However, this was less effective when TPS-G-CPA was used. Nevertheless, the mechanical properties of both blends were similar in terms of tensile strength. The plasticizing effect of CPA was confirmed by an increase in elongation at break of the LDPE/TPS-G-CPA blends compared to those with TPS-G. Acceptable fire behavior was achieved by combining the starch-based FR with a commercial flame retardant (FR69), with the most promising blends being 47.5 LDPE/38 TPS-G/9.5 FR69 and 47.5 LDPE/19 TPS-G-CPA/28.5 FR69. These blends had a similar LOI (about 28%) to that of reference blend containing only the commercial flame retardant (50 LDPE/50 FR69). CPA not only imparts higher plasticity to the starch, but also improves the charring ability, flame retardancy, and effective combustion component content of the blends, as well as a 43% reduction in fire growth index compared to the reference blends, suggesting that it may contribute to lower fire spread in the event of a fire. These results indicate that starch plasticized in the presence of water, glycerol and CPA has a promising future as a safe and cost-effective flame retardant for LDPE formulations.

## Figures and Tables

**Table 1:** Composition of the two types of TPS prepared.

Sample	Potato Starch (wt%)	Glycerol (wt%)	Distilled Water (wt%)	CPA (wt%)
TPS-G	62.5	25.0	12.5	--
TPS-G-CPA	50.0	20.0	10.0	20.0

**Table 2:** Composition of LDPE/TPS-G(-CPA)/FR69 blends compatibilized with PE-*g*-MA and prepared via melt blending. LDPE/FR69 was used as reference material.

Sample	LDPE (wt%)	TPS g or TPS-G-CPA (wt%)	FR69 (wt%)	PE-*g*-MA (wt%)
50 LDPE/50 FR69	50	0	50	0
47.5 LDPE/9.5 TPS-G (or TPS-G-CPA)/38 FR69	47.5	9.5	38	5
47.5 LDPE/19 TPS-G (or TPS-G-CPA)/28.5 FR69	47.5	19	28.5	5
47.5 LDPE/28.5 TPS-G (or TPS-G-CPA)/19 FR69	47.5	28.5	19	5
47.5 LDPE/38 TPS-G (or TPS-G-CPA)/9.5 FR69	47.5	38	9.5	5

**Table 3:** Cone calorimetry results of selected LDPE/flame retardant blends.

Sample	50 LDPE/50 FR69	47.5 LDPE/38 TPS-G/9.5 FR69	47.5 LDPE/19 TPS-G-CPA/28.5 FR69
pHRR (kW/m^2^)	651.56	621.48	663.01
t_pHRR_ (s)	453.3	325.0	808.0
THR (MJ/m^2^)	25.56	34.25	32.27
TTI (s)	46.3	22.3	27.7
EHC (MJ/kg)	14.97	14.12	10.00
MARHE (kW/m^2^)	184.83	229.08	200.19
FPI (m^2^/kW)	0.07	0.04	0.04
FGI (kW/m^2^·s)	1.44	1.91	0.82

pHRR: peak of heat release rate; t_pHRR_: time to peak of heat release rate; THR: total heat released; TTI: time to ignition; EHC: effective heat of combustion; MARHE: maximum average rate of heat emission, FPI: fire performance index; FGI: fire growth index.

**Table 4:** Thermal properties of the blends obtained by means of TG analysis.

Sample	*T*_5wt%_ (°C) ^a^	*T*_10wt%_ (°C) ^b^	CR_600°C_ (%) ^c^
50 LDPE/50 FR69	337.9	349.4	3.0
47.5 LDPE/38 TPS-G/9.5 FR69	191.7	264.3	6.5
47.5 LDPE/19 TPS-G-CPA/28.5 FR69	230.6	281.2	10.5

^a^ *T*_5wt%_: temperature at 5% weight loss; ^b^ *T*_10wt%_: temperature at 10% weight loss; ^c^ CR_600°C_: char residue at 600 °C.

## Data Availability

Not applicable.

## Supplementary Materials

The following supporting information can be downloaded at: https://www.mdpi.com/article/10.3390/polym15204078/s1, Figure S1: The 400 MHz ^1^H NMR spectrum of CPA in D_2_O; Table S1: Composition of LDPE/TPS-G(-CPA) blends compatibilized with PE-*g*-MA and prepared by melt blending. LDPE was used as reference material (100LDPE code); Figure S2: Preliminary burning tests conducted in the laboratory and mimicking the UL94 test for (a) LDPE/TPS-G blends and (b) LDEP/TPS-G-CPA blends. LDPE was used as reference material.
