# Supplementary material for: Replacing Harmful Flame Retardants with Biodegradable Starch-Based Materials in Polyethylene Formulations
Source: Polymers (Basel). 2023 Oct 13;15(20):4078. doi: 10.3390/polym15204078 (PMC10610673; doi:10.3390/polym15204078)
Supplement: Supplementary file 1 [file polymers-15-04078-s001.zip › polymers-2568269-supplementary.pdf]

Supporting Information

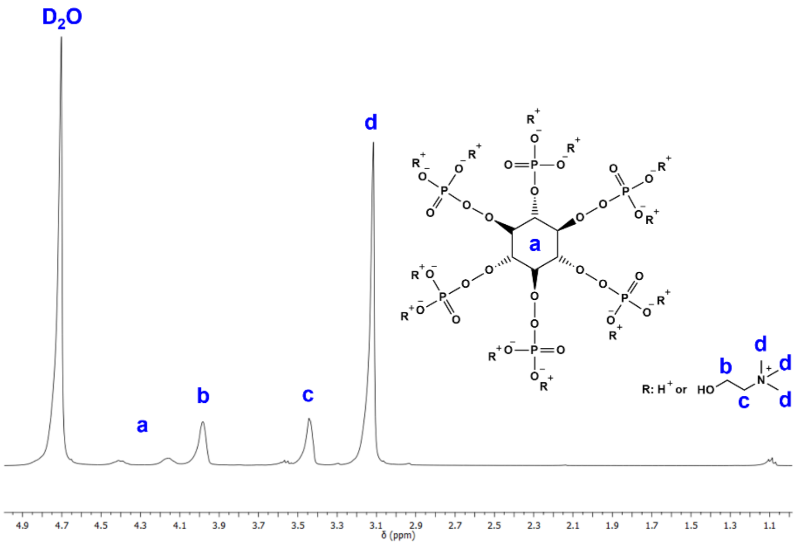

Figure S1. 400 MHz ^{1}H NMR spectrum of CPA in D_2O.

Table S1. Composition of LDPE/TPS-G(-CPA) blends compatibilized with PE-*g*-MA and prepared by melt blending. LDPE was used as reference material (100LDPE code).

Sample code	LDPE (wt%)	TPS-G (wt%)	TPS-G-CPA (wt%)	PE-*g*-MA (wt%)
100 LDPE	100.0	0.0	0.0	0.0
21 LDPE/74 TPS-G/5 PE-*g*-MA	21.0	73.75	0.0	5.0
73 LDPE/22 TPS-G/5 PE-*g*-MA	73.0	21.25	0.0	5.0
47.5 LDPE/47.5 TPS-G/5 PE-*g*-MA	47.5	47.5	0.0	5.0
73 LDPE/22 TPS-G-CPA/5 PE-*g*-MA	73.0	0	22.0	5.0
47.5 LDPE/47.5 TPS-G-CPA/5 PE-*g*-MA	47.5	0	47.5	5.0

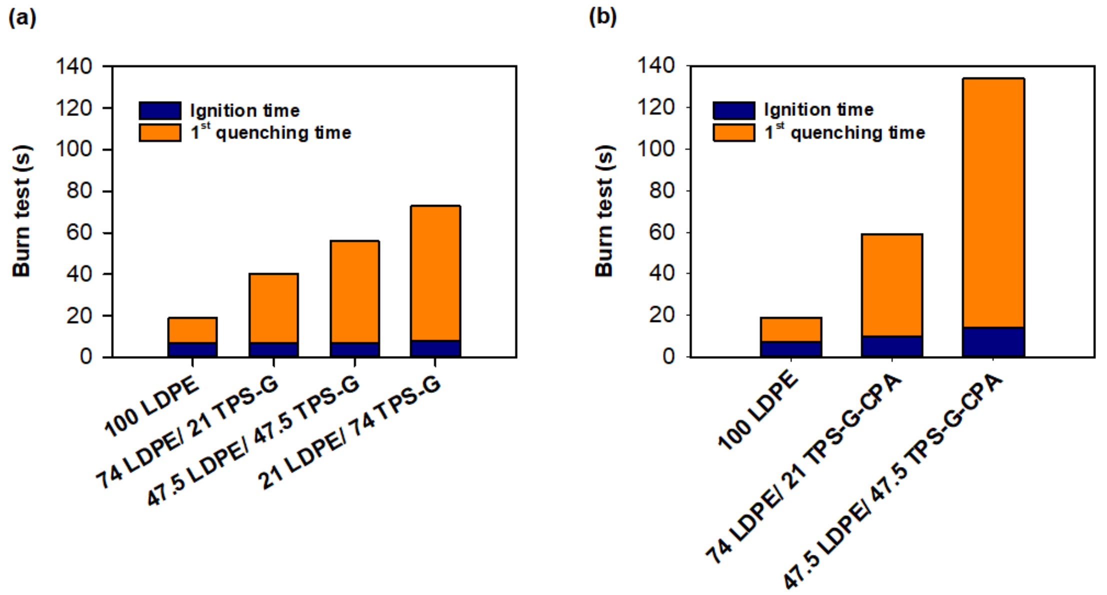

Figure S2. Preliminary burning tests conducted in the laboratory and mimicking the UL94 test for (a) LDPE/TPS-G blends and (b) LDEP/TPS-G-CPA blends. LDPE was used as reference material.
